# Transcriptome analysis provides *StMYBA1* gene that regulates potato anthocyanin biosynthesis by activating structural genes

**DOI:** 10.3389/fpls.2023.1087121

**Published:** 2023-01-20

**Authors:** Xijuan Zhao, Huiling Zhang, Tengfei Liu, Yanan Zhao, Xinxi Hu, Shengxuan Liu, Yuan Lin, Botao Song, Changzheng He

**Affiliations:** ^1^ Engineering Research Center for Germplasm Innovation and New Variety Breeding of Horticultural Crops, Key Laboratory for Vegetable Biology of Hunan Province, Hunan Agricultural University, Changsha, China; ^2^ Key Laboratory of Potato Biology and Biotechnology, Ministry of Agriculture and Rural Affairs, National Key Laboratory for Germplasm Innovation and Utilization of Horticultural Crops, College of Horticulture and Forestry Sciences, Huazhong Agricultural University, Wuhan, China; ^3^ College of Horticulture and Plant Protection, Henan University of Science and Technology, Luoyang, China

**Keywords:** potato, anthocyanin biosynthesis, light-induced, *StMYBA1*, transcriptional activation

## Abstract

Anthocyanin biosynthesis is affected by light, temperature, and other environmental factors. The regulation mode of light on anthocyanin synthesis in apple, pear, tomato and other species has been reported, while not clear in potato. In this study, potato RM-210 tubers whose peel will turn purple gradually after exposure to light were selected. Transcriptome analysis was performed on RM-210 tubers during anthocyanin accumulation. The expression of *StMYBA1* gene continued to increase during the anthocyanin accumulation in RM-210 tubers. Moreover, co-expression cluster analysis of differentially expressed genes showed that the expression patterns of *StMYBA1* gene were highly correlated with structural genes *CHS* and *CHI*. The promoter activity of *StMYBA1* was significantly higher in light conditions, and StMYBA1 could activate the promoter activity of structural genes *StCHS*, *StCHI*, and *StF3H*. Further gene function analysis found that overexpression of *StMYBA1* gene could promote anthocyanin accumulation and structural gene expression in potato leaves. These results demonstrated that *StMYBA1* gene promoted potato anthocyanin biosynthesis by activating the expression of structural genes under light conditions. These findings provide a theoretical basis and genetic resources for the regulatory mechanism of potato anthocyanin synthesis.

## 1 Introduction

Potato (*Solanum tuberosum* L.) is the most important non-cereal crop worldwide. It has been found that pigmented potato cultivars are rich in anthocyanin (Fossen and Andersen, 2000) addition to their colorful appearance and enhanced flavor, pigmented potato tubers also exhibit high antioxidant activity. It was found that the oxidation activity of red and purple potato tubers was 2–3 times higher than that of white tubers (Brown, 2005). Therefore, consuming pigmented potatoes can also protect against some cancers and cardiovascular diseases (He and Giusti, 2010; Crowe et al., 2011).

Anthocyanins are synthesized through the flavonoid pathway, which includes both regulatory and structural genes. The structural genes can be divided into two groups, early biosynthetic genes (EBGs) and late biosynthetic genes (LBGs), based on the catalytic substrate. The EBGs include chalcone synthase (*CHS*), chalcone isomerase (*CHI*), and flavanone 3-hydroxylase (*F3H*) which catalyze the formation of colorless dihydroflavonol compounds. The LBGs consist of flavonoid 3′-hydroxylase (*F3′H*), flavonoid 3′,5′-hydroxylase (*F3′5′H*), dihydroflavonol 4-reductase (*DFR*), anthocyanin synthase (*ANS*), and flavonoid 3-O-glucosyltransferase (*3GT*), usually catalyzing the formation of colored anthocyanins (Baudry et al., 2004; Liu et al., 2018). The regulatory genes modulating anthocyanin biosynthesis are mainly through the well-documented MBW complexes which formed by MYB, bHLH, and WD40 (Allan et al., 2008; Lloyd et al., 2017). MYB transcription factors (TFs) are believed to play crucial roles in regulating anthocyanin biosynthetic genes (Lai et al., 2013), as well as, many MYB TFs have been demonstrated to be activators of the anthocyanin accumulation from many species, such as Arabidopsis (Borevitz et al., 2000; Gonzalez et al., 2008), apple (Espley et al., 2007; Jiang et al., 2021), and pear (Yao et al., 2017). In potato, the MYB gene *StAN2* can promote anthocyanin accumulation in tubers by regulating the structural genes (Jung et al., 2009; Liu et al., 2016). Another two MYB genes, *StMYBA1* and *StMYB113*, could promote anthocyanin biosynthesis in tobacco leaves (Liu et al., 2016; Liu et al., 2017), and *StMYBA1* promoted anthocyanin synthesis, which requires light (Liu et al., 2017). However, it is unclear whether they can promote anthocyanin biosynthesis in potato.

Anthocyanins biosynthesis is not only affected by genes, but also regulated by many environmental factors, such as low temperatures, salt, drought, and light (Cominelli et al., 2008; Catala et al., 2011; Li et al., 2017). In purple kale, the anthocyanin accumulation is induced by low temperature, the total content of anthocyanin under low temperature was about 50-fold higher than that of the plants grown in the greenhouse (Zhang et al., 2012). Light is an important environmental factor affecting anthocyanin biosynthesis according to light quality and light intensity. Also, the effect of light on anthocyanin accumulation has been reported in tomato (Wang et al., 2021), eggplant (Li et al., 2017), apple (Li et al., 2012; Chen et al., 2019), pear (Liu et al., 2019), grape (Azuma et al., 2012) and many other species. The light regulation mechanism of anthocyanin biosynthesis is that the light response proteins such as COP1, and HY5, inhibit or promote anthocyanin biosynthesis by degrading or activating MBW members, mainly MYB TFs. *PAP1* and *PAP2*, two members of MYB genes, are required for anthocyanin accumulation in arabidopsis. In dark-grown plants, the photoresponsive protein COP1 interacts with PAP1 and PAP2 and degrades them, so there is no accumulation of anthocyanin. However, anthocyanin is biosynthesized when COP1 is degraded under light conditions (Maier et al., 2013). In apples, PdHY5 regulates the *MdMYB10* gene at transcriptional and post-transcriptional levels under light conditions, thereby activating the expression of structural genes to promote anthocyanin synthesis (Espley et al., 2007; An et al., 2017). Moreover, MYB genes related to light-stimulated anthocyanin synthesis were identified in pear (Wang et al., 2020), buckwheat (Yao et al., 2020) and other species.

In our previous study, we found that the tubers of potato population F_1_RM are yellow peel yellow flesh, but the peel of the tubers will turn purple gradually after exposure to light. In this study, the tuber anthocyanin content and transcriptome sequencing analyze were further applied to these light-sensitive F_1_RM tubers, to explore the key regulatory genes of light-induced potato anthocyanin biosynthesis and reveal its regulatory mechanism.

## 2 Methods

### 2.1 Plant materials

The potato population F_1_RM was obtained by crossing with the heterozygous diploid yellow peel yellow flesh potato RH (*S. tuberosum*) as the female parent and the yellow peel yellow flesh diploid inbred clone M6 (*S. chacoense*) as the male parent. F_1_RM tubers are also yellow peel yellow flesh, but the peel of the tubers will turn purple gradually after exposure to light. Among them, the tubers of line RM-210 were selected to study the regulation of anthocyanin accumulation during light exposure. The RM-210 plants were grown in 24-cm-diameter plastic pots in greenhouse with 16 h light per day supplied by lamps. For about 80 days after planting, the mature tubers were harvested and dark storage for 10 days at 20°C. Then they were incubated under the continuous light intensity of 2000 LX, and samples were collected at 0, 6, 12, 36, 48, and 120 h. Five tubers were used for sampling at each time point, and the peels (1 mm thick) were collected and stored at –70°C for molecular and biochemical analyses.

*Nicotiana benthamiana* L. for dual luciferase assays was grown under greenhouse conditions until 5–6 leaves were available for *Agrobacterium* infiltration.

### 2.2 Anthocyanin extraction and content analysis

Anthocyanin mixtures were extracted according to the agricultural standard of China (NY/T 2640-2014). 100mg powder was extracted with 1.0 ml 1% formic acid methanol overnight at 4°C. Extracts were centrifuged at 10,000 ×g for 10 min and filtrated with 0.22-μm microporous membrane. Anthocyanin quantification was measured by High Performance Liquid Chromatography (HPLC) as a previous description (Zhou et al., 2008). The anthocyanin standard consisted of peonidin, delphinidin, malvidin, petunidin, pelargonidin, and cyanidin dissolved in methanol.

### 2.3 RNA isolation and library construction

Total RNA was isolated according to previous description (Zhang et al., 2022). The RNA integrity was assessed using the Fragment Analyzer 5400 (Agilent Technologies, CA, USA). Sequencing libraries were generated using NEBNext^®^ Ultra™ RNA Library Prep Kit for Illumina^®^ (NEB, USA) in accordance with the manufacturer’s recommendations and index codes were added to attribute sequences to each sample.

### 2.4 Sequence data filtering, *de novo* assembly, and annotation

The original files obtained from the Illumina platform are transformed into raw data by base calling. The raw reads were trimmed by removing adapters, reads containing ploy-N more than 5% and low-quality sequences. Raw sequences were transformed into clean reads after processing. Then the clean reads were mapped to the transcriptome of the potato DM reference genomes (6.1 version, http://spuddb.uga.edu/dm_v6_1_download.shtml) with Salmon software, version 1.3.0 (Patro et al., 2017).

### 2.5 Differentially expressed gene analyses

Transcripts Per Kilobase Million (TPM) were used to estimate gene expression level. The R package DESeq2 was used to perform the differential gene expression among different samples. Thresholds for significantly differential expression were false discovery rate (FDR) ≤ 0.05 and fold change ≥2 or ≤ 0.5. The KEGG pathway enrichment analysis of differentially expressed genes (DEGs) was performed by TBtools at the adjusted p <  0.05 by using a hypergeometric test (Chen et al., 2020). The cluster analysis was performed as described previously (Guo et al., 2016).

### 2.6 Reverse transcription quantitative real-time PCR

Reverse transcription quantitative real-time PCR (RT-qPCR) primers were designed with the NCBI Primer-BLAST and listed in **Table 1**. The RT-qPCR was performed on a CFX96™ real-time PCR system (Bio-Rad, USA) with the TransStart Top Green qPCR SuperMix kit (Transgen, Beijing, China). The potato housekeeping genes *ef1α* (GenBank: AB061263) and *actin* (GenBank: XM_006345899.2) were selected as internal reference genes. Relative expression of the individual gene was calculated with calculated multiple internal control method (Vandesompele et al., 2002).

**Table 1:** The primer sequences for qPCR and constructing vectors.

Primer name	Gene ID	Forward primer (5’-3’)	Reverse primer (5’-3’)	Used for
StMYBA1	Soltu.DM.10G020840.1	CCTCAACCTCGGAACTTCTCA	TCCTTGCAACGTTTGTCGTC	qRT-PCR
StCHS1	Soltu.DM.09G028560.1	CGGCCGCTATCATTATGGGT	TCCATCGATAGCACCTTCGC	
StCHS2	Soltu.DM.05G023610.1	ACTTTTCGTGGCCCAAGTGA	AAGGCCTTTCGACTTCTGGT	
StCHI1	Soltu.DM.05G001950.1	ATGCAGAGAGGGAGGCCATT	CGTCAATGATCCAAGCGGTG	
StCHI2	Soltu.DM.07G023150.1	GCCGTCTTTCAAGGGAAACC	ACGTCGAAGAGAGCTGATGC	
StPAL1	Soltu.DM.09G005700.1	CAGCTGCACCTACCCTTTGA	ACAGGGTTGCCACTTTCAAGA	
StPAL2	Soltu.DM.10G020990.1	TGGCAGGCCTAATTCCAAGG	TACCGCGAGCACATTAGCAT	
StC4H1	Soltu.DM.06G032850.1	AAGCTTCCGTACCTTCAGGC	AGGTTTCTTCCAGTGAGCGG	
StC4H2	Soltu.DM.06G032860.1	AAGCTTCCGTACCTTCAGGC	AGGTTTCTTCCAGTGAGCGG	
St4CL	Soltu.DM.03G020790.1	GCTTGATTACAGGGGTGGCT	AGCTCCAACAGCGCACTAAT	
StMYB48	Soltu.DM.11G026620.2	ACCAGGGCGTACTGATAACG	AAGAGCTTGATTCCACCCGT	
StB3	Soltu.DM.09G002750.1	GAGCAATGCCGAACCAAACA	ATCCTGGTTGTGGCTCGATG	
StHSF2	Soltu.DM.07G011880.1	TAACAGCAGCCCAGAGATGG	GCACTCGGATGGGTTTGAGA	
pSAK-StMYBA1	Soltu.DM.10G020840.1	TAGTGGATCCAAAGAATTCCGTTGGGAGTTAGGAAAGG	CGAGAAGCTTTTTGAATTCTTAATTAAGTAGATTCCATAAG	Construction vector
Prom-StMYBA1	Soltu.DM.10G020840.1	TAGTGGATCCAAAGAATTCCGTTGGGAGTTAGGAAAGG	CGAGAAGCTTTTTGAATTCTTAATTAAGTAGATTCCATAAG	
Prom-StCHS	Soltu.DM.05G023610.1	ATAGGGCGAATTGGGTACCCCTACTTAACAATCAAACACAACAA	TTTTTGGCGTCTTCCATGGCGTGTTTTTTTTTTTACTAAGATTT	
Prom-StCHI	Soltu.DM.07G023150.1	ATAGGGCGAATTGGGTACCTCACTAACCTGAGAAGTAGGACGAG	TTTTTGGCGTCTTCCATGGATCAGTTGTATTATTACCAGAAGAGGAG	
Prom-StF3H	Soltu.DM.02G023850.1	ATAGGGCGAATTGGGTACCTTGACATGTTTTTTTTTTAGCTAGG	TTTTTGGCGTCTTCCATGGGTTCAAAAGAGTTATGAGGTGCC	
Prom-StF3’H	Soltu.DM.03G029340.1	ATAGGGCGAATTGGGTACCAAAAAAGGCTAGTCAAATGGGATAA	TTTTTGGCGTCTTCCATGGCGGGCCATTGATGCAGTG	
Prom-StF3’5’H	Soltu.DM.11G020990.1	ATAGGGCGAATTGGGTACCATGTAAAAAATACGATAACAAAAAGT	TTTTTGGCGTCTTCCATGGCAACATGTGGCATTGAACCT	
Prom-StANS	Soltu.DM.08G026700.1	ATAGGGCGAATTGGGTACCTATTGTGACTTTAGCTTTCATGATC	TTTTTGGCGTCTTCCATGGTGTTACGCGGAGTACTTATTTAGA	
Prom-StDFR	Soltu.DM.02G024900.2	ATAGGGCGAATTGGGTACCCGCTATGTTATTGTTAAGGGTG	TTTTTGGCGTCTTCCATGGCAGAAATGAGAGGAAAAAAGAGTC	
Prom-StGST	Soltu.DM.02G020850.1	ATAGGGCGAATTGGGTACCCTTCAGTTCAAAAATAGTTTCCTCT	TTTTTGGCGTCTTCCATGGCTTTTTTTTTTCTTGTGAAATCC	

### 2.7 Construction of expression vectors and transient assays

According to the Soltu.DM.10G020840.1 sequence from the Potato Genome Database, the open reading frame (ORF) of the *StMYBA1* was amplified from the cDNA of RM-210 tuber peel. The ORF of *StMYBA1* was cloned into the pSAK-277 vector through *Eco*RI restriction sites, and the recombinant plasmid pSAK-StMYBA1 was sequenced (Sangon Biotech, Shanghai, China). The primer sequences pSAK-StMYBA1-F/R were listed in **Table 1**.

The promoter sequences of *StMYBA1*, *StCHS*, *StCHI*, *StF3H*, *StF3’H*, *StDFR*, *StF3’5’H*, *StANS* and *StGST* were isolated from M6 (the tubers peel will turn purple gradually after exposure to light) and cloned into the pGreenII0800-LUC vector with the *Kpn*I and *Nco*I restriction sites. The primer sequences were listed in **Table 1**. Dual-luciferase assays were performed in tobacco, as previously described (Liu et al., 2016; Zhang et al., 2022), and the pGreenII0800-LUC vector without a promoter was used as a negative control. pSAK-StMYBA1 was infiltrated into tobacco leaves with pGreenII0800-StCHS, pGreenII0800-StCHI, pGreenII0800-StF3H, pGreenII0800-StF3’H, pGreenII0800-StF3’5’H, pGreenII0800-StDFR, pGreenII0800-StGST and pGreenII0800-StANS in a second *Agrobacterium* strain, respectively. To analyze the light response of the *StMYBA1* gene promoter, tobacco leaves were infected with *Agrobacterium* containing fusion vectors pGreenII0800-StMYBA1, and the pGreenII0800-LUC vector without a promoter was selected as a negative control. After infiltration, the tobacco plants were kept in the dark for 12 h. Then some of the plants were moved to the culture chamber with 16 h of light per day, and the other plants were still in the dark. The leaves were sampled after three days of infiltration and stored at –70°C. The LUC and REN activities were tested using the Dual-Luciferase^®^ reporter Assay System (Promega, USA).

### 2.8 Transformation of potato

The overexpression (OE) vector pSAK-StMYBA1 was transformed into the potato diploid clone AC142 as previously (Si et al., 2003; Zhang et al., 2014). The transgenic plants and untransformed control were grown in 12-cm-diameter plastic pots in the greenhouse. For one month after planting, the leaves were sampled, frozen in liquid nitrogen, and stored at –70°C until use.

## 3 Results

### 3.1 Light-induced anthocyanin accumulation in tuber peel of potato

**Figure 1:**
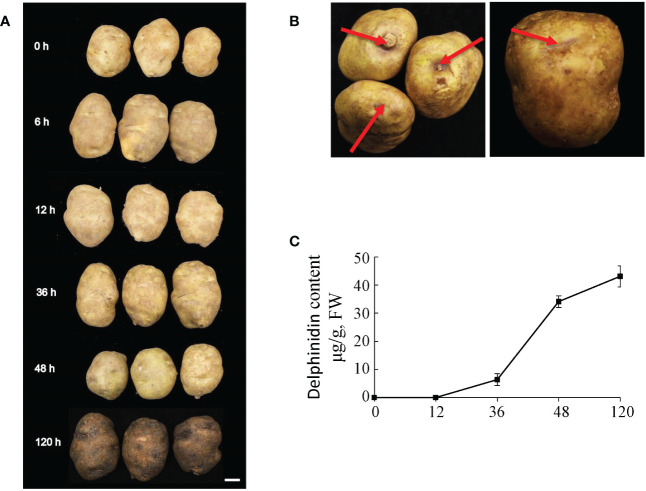
Tuber phenotype and anthocyanin content of RM-210 tubers. **(A)** The phenotype of RM-210 tubers during light treatment. **(B)** The phenotype of the RM-210 tubers exposed to light 48 h. **(C)** Delphinidin content of RM-210 tubers.

Tubers of potato population F_1_RM are yellow peel yellow flesh, while the peel of the tubers will turn purple gradually after exposure to light (**Figure 1A**). Among them, the tubers of line RM-210 were selected to study the regulation of anthocyanin accumulation during light exposure. Purple can be seen at the top of the RM-210 tubers at 48 h light exposure (**Figure 1B**), and the whole tuber peels appear to be purple at 120 h (**Figure 1A**). Thus, the time points of sampling for anthocyanin content and RNA-seq analysis were 0, 6, 12, 36, 48, and 120 h.

The HPLC chromatograms of the extracts from the peel of tubers are shown in **Figure S1**, and one peak was detected at approximately 0.91 min. According to the comparison analysis with the standard, the component represented by the spectrogram is delphinium. The results showed that delphinium could not be detected in the tuber peel with 0 and 12 h light exposure, and the content of delphinium in the tuber peel for 36, 48, and 120 h was 6.36, 34.08, and 43.07 mg/kg, respectively (**Figure 1C**). This indicates that light can promote the accumulation of delphinium in RM-210 tuber peel and show purple.

### 3.2 Analysis of differentially expressed genes during anthocyanin accumulation

**Figure 2:**
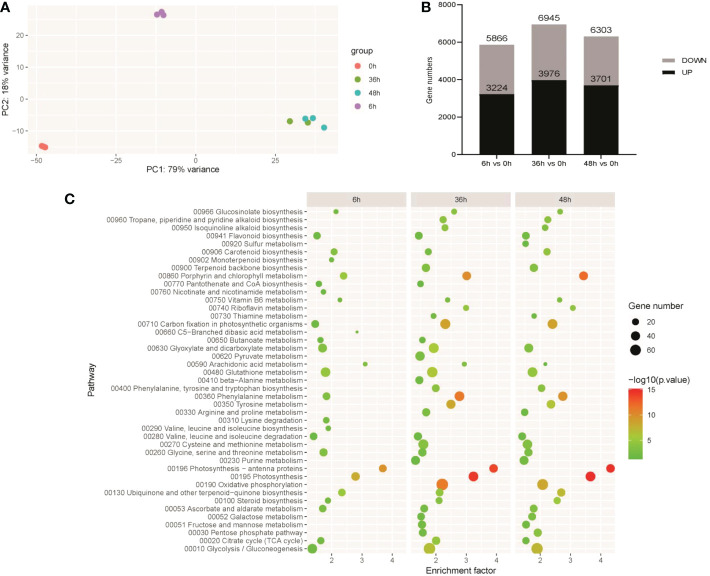
Overview of transcriptome analysis. **(A)** Correlation analysis of RNA-seq data of different treatments. **(B)** The number of differentially expressed genes (DEGs) in each comparison. **(C)** Kyoto Encyclopedia of Genes and Genomes (KEGG) enrichment analysis of DEGs in each comparison.

**Figure 3:**
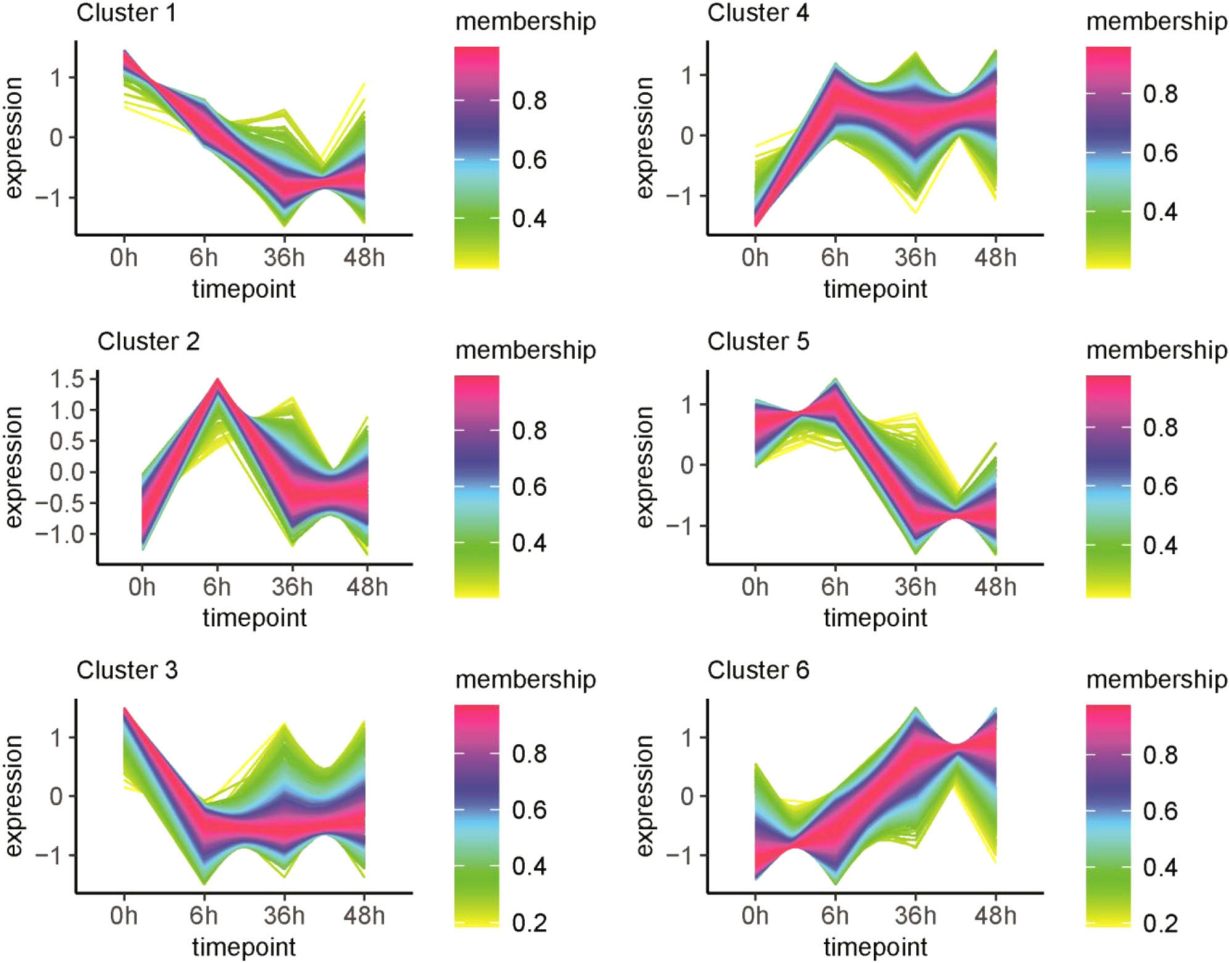
Cluster analysis of gene expression patterns in RM-210 tubers at different time points of light treatment. The Y-axis represents the deviation of the gene expression. The X-axis represents the sampling time points.

To understand the key pathways and regulatory genes in light regulation of anthocyanin biosynthesis, transcriptomics based on RNA-seq was performed on RM-210 tuber peel response to light. mRNA-sequencing libraries were generated from tuber peel samples obtained at 0, 6, 36, and 48 h light exposure, respectively, and except for two replicates from samples obtained at 36 h, all other samples had three replicates. The relationship among the samples was analyzed by the Principle Component Analysis (PCA) approach, which shows that the three replicates of the same sample are polymerized together with good repeatability (**Figure 2A**). Therefore, it was suggested that the RNA-seq data was suitable for further differential gene expression (DGE) analysis. To better understand the overall expression patterns of the transcripts in four samples, clustering analysis of the expression patterns in different samples was performed using the R package “TCseq”. There were six clusters in total from the four samples (**Figure 3**). Interesting, the expression levels of genes from cluster 2 were higher at 6h than other samples, indicating that the genes in this cluster were the early light response. The expression of genes from class 6 increased with the light duration and anthocyanin accumulation, indicates that the function of these genes may related to anthocyanin accumulation.

To explore the genes contributing to anthocyanin biosynthesis in response to light, comparisons 6h vs 0h, 36h vs 0h, and 48h vs 0h were made. Based on fold change ≥ 2 and FDR < 0.01 criteria, 3224, 3976, and 3701 genes up-regulated were identified in comparisons 6h vs 0h, 36h vs 0h, and 48h vs 0h respectively, and 2642, 2969, and 2602 genes respectively were significantly down-regulated (**Figure 2B**). The list of DEGs in each comparison is shown in **Table S1**, and the KEGG pathway enrichment analysis was performed. There were 26 pathways enriched in 6h vs 0h, the top 5 of which were photosynthesis-antenna proteins, photosynthesis, porphyrin and chlorophyll metabolism, ubiquinone and other terpenoid-quinone biosynthesis, and glutathione metabolism. There were 37 pathways enriched in 36h vs 0h, the top 5 of which were photosynthesis, photosynthesis-antenna proteins, phenylalanine metabolism, oxidative phosphorylation, and porphyrin and chlorophyll metabolism. In 48h vs 0h, there were 34 pathways enriched, of which the first 5 were photosynthesis, photosynthesis-antenna proteins, porphyrin and chlorophyll metabolism, phenylalanine metabolism, and carbon fixation in photosynthetic organisms (**Figure 2C**).

### 3.3 Identification of transcription factors related to anthocyanin biosynthesis

**Figure 4:**
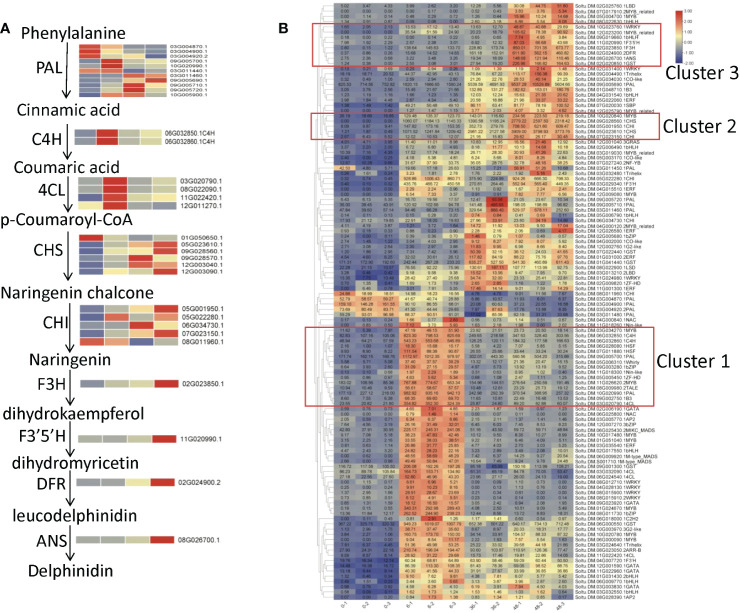
Co-expression analysis of structural genes and transcription factors in DEGs. **(A)** Anthocyanin biosynthesis pathway. **(B)** Co-expression cluster analysis of structural genes and transcription factors. The red boxes mark transcription factors that have a high correlation with the expression of key structural genes.

Delphinidin in potato is mainly synthesized by structural genes (**Figure 4A**) and regulated transcription factors. The expression levels of most structural genes in the four samples were different. For example, the expression of most *PAL* genes were high at 0h or 6h, *C4H* and *4CL* genes were high at 6h, *CHS* and *CHI* genes were high at 36h or 48h, and the expression of *F3H*, *F3’5’H*, *DFR*, and *ANS* were high at 48h (**Figure 4A**). This result indicated that the expression of these early biosynthesis genes of anthocyanin is earlier than that of late biosynthesis genes.

To further explore the key transcription factors regulating structural genes, 114 differentially expressed structural genes, and TFs (**Table S2**) were subjected to co-expression cluster analysis. The results showed that there were three clusters with a strong correlation between major structural genes and TFs (**Figure 4B**). The structural genes *PAL* (Soltu.DM.09G005700.1, Soltu.DM.10G020990.1), *C4H* (Soltu.DM.06G032850.1, Soltu.DM.06G032860.1), *4CL* (Soltu.DM.03G020790.1) and MYB transcription factors (Soltu.DM.03G034270.1, Soltu.DM.11G026620.2), bZIP (Soltu.DM.09G003280.1) and B3 (Soltu.DM.09G002750.1) transcription factors were included in cluster 1. Above structural genes, *PAL*, *C4H* and *4CL* had the highest expression in the sample exposed to light for 6 h, so it was speculated that the transcription factors in cluster 1 might be early light response factors. Cluster 2 included two *CHS* genes (Soltu.DM.09G028560.1, Soltu.DM.05G023610.1), two *CHI* genes (Soltu.DM.05G001950.1, Soltu.DM.07G023150.1) and one *MYB* gene (Soltu.DM.10G020840.1, *StMYBA1*), whose expression level is gradually increased with the extension of treatment time. And structural genes *F3H* (Soltu.DM.02G023850.1), *F3’5’H* (Soltu.DM.11G020990.1), *DFR* (Soltu.DM.02G024900.2), *ANS* (Soltu.DM.08G026700.1), *GST* (Soltu.DM.02G020850.1), and MYB (Soltu.DM.12G023200.1), bHLH (Soltu.DM.09G019660.1, *StAN1*), and WRKY (Soltu.DM.10G023760.1, *StWRKY13*) transcription factors were included in cluster 3. Expression levels of all the five structural genes in cluster 3 increased rapidly in the sample exposed to light for 48 h, indicating that the transcriptional factors associated with them may also function at the late stage of anthocyanin biosynthesis. To assess the repeatability of the sequencing data, the genes in cluster1 and cluster2 were selected to determine their expression levels using RT-qPCR. The results showed that most of them had a similar expression trend with transcriptome analysis results (**Figure S2**).

### 3.4 Regulation of *StMYBA1* gene on structural genes

**Figure 5:**
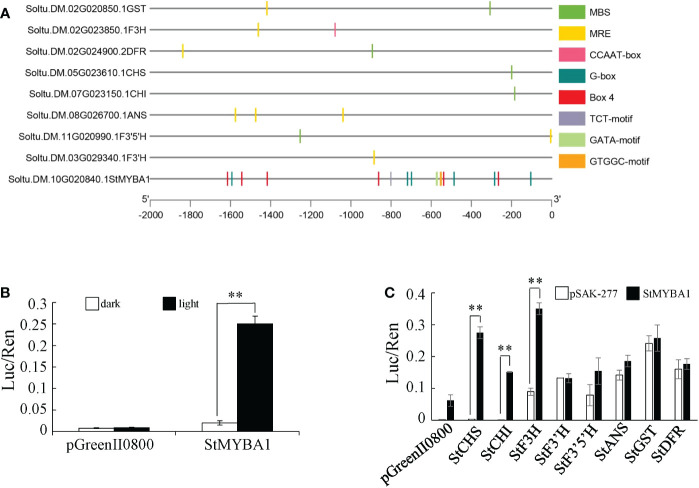
Light Response analysis of *StMYBA1* Gene and its regulation on structural genes. **(A)** Distribution of main core elements of *StMYBA1* and structural gene promoters. **(B)** Activity analysis of *StMYBA1* promoter under light and dark conditions. **(C)** Regulation of *StMYBA1* on structural genes related to anthocyanin biosynthesis. Significant differences (LSD) between means of the dark and light treatments, and pSAK-277 and StMYBA1 are indicated by asterisks (** p < 0.01).

The *StMYBA1* gene in cluster 2 was previously confirmed to promote anthocyanin accumulation in tobacco leaves, and light is required for its function on anthocyanin accumulation (Liu et al., 2017). Analysis of the promoter sequences of *StMYBA1* showed that it contained multiple cis-acting regulatory elements involved in light responsiveness, such as Box4 (ATTAAT) and G-box (CACGTC/CACGTC/TACGTG) (**Figure 5A**, **Table S3**). To further analyze the response of the *StMYBA1* promoter to light, tobacco leaves were infected with *Agrobacterium* containing fusion vectors pGreenII0800-StMYBA1. The dual luciferase activity in tobacco leaves was evaluated after 3d incubation in darkness or light. The result showed that the promoter activity of *StMYBA1* in light conditions was significantly higher than that in dark, and no differences were observed in control (**Figure 5B**), suggesting that the activity of *StMYBA1* promoter could be induced by light.

The 2Kb promoter sequences of key structural genes in potato anthocyanin biosynthesis (*StCHS*, Soltu.DM.05G023610.1; *StCHI*, Soltu.DM.07G023150.1; *StF3H*, Soltu.DM.02G023850.1; *StF3’H*, Soltu.DM.03G029340.1; *StF3’5’H*, Soltu.DM.11G020990.1; *StDFR*, Soltu.DM.02G024900.2; *StANS*, Soltu.DM.08G026700.1; *StGST*, Soltu.DM.02G020850.1) were selected. The sequence analyzing showed that each of the structural gene promoter sequences contained at least one MYB binding elements (**Figure 5A**, **Table S3**). Therefore, transient luciferase assays in tobacco were used to test the activation of these structural gene promoters by StMYBA1. The results showed that compared to the negative control, StMYBA1 can significantly activate the promoters of *StCHS*, *StCHI*, and *StF3H*, but not the promoters of the other five structural genes (**Figure 5C**).

### 3.5 Regulation of *StMYBA1* gene on anthocyanin biosynthesis in potato

**Figure 6:**
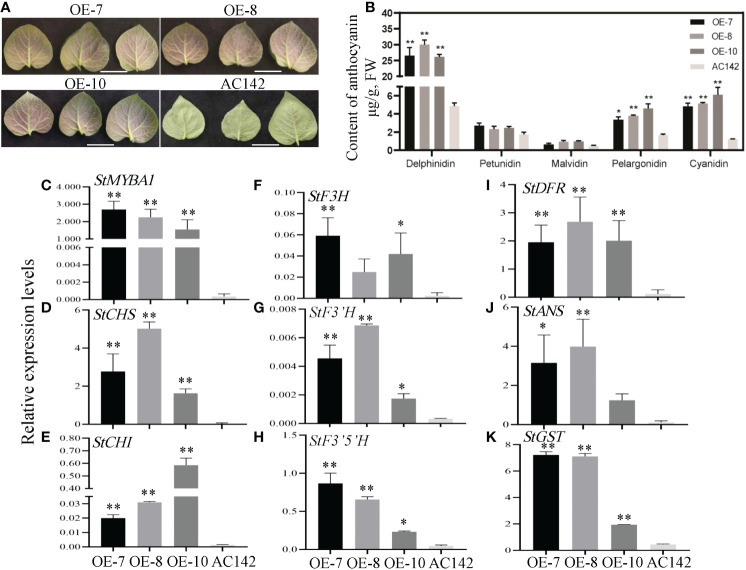
Regulation of *StMYBA1* gene on anthocyanin biosynthesis in potato. **(A)** Representative leaves images of StMYBA1-overexpressed transgenic lines (OE-7, OE-8, and OE-10) and the wild type (AC142). Bars=2 cm. **(B)** The anthocyanin content in the *StMYBA1* transgenic leaves. **(C–K)** The relative expression of *StMYBA1* and structural genes in the transgenic leaves. Significant differences (LSD) between means of the transgenic lines and wild type are indicated by asterisks (* p < 0.05, ** p < 0.01).

To explore the function of *StMYBA1* in potato anthocyanin biosynthesis, the potato genotype AC142 was transformed with expression vector pSAK-StMYBA1 (**Figure S3A**). After rooting screening (**Figure S3B**) and gene expression analysis, nineteen overexpression transgenic lines with increased *StMYBA1* transcripts were generated (**Figure S3C**). Three independent transgenic lines (OE-7, OE-8, and OE-10) were selected for function analysis. The transgenic lines showed normal plant morphology under greenhouse growing conditions comparing to wild type (**Figure S3D**). The leaf phenotype of transgenic lines was observed after 30 days of planting. Leaves of transgenic lines were purple, whereas those of wild-type were green (**Figure 6A**). Anthocyanin content in transgenic leaves was analyzed. The contents of delphinidin, pelargonidin, and cyanidin in the leaves of the transgenic lines were 5.37–6.15, 2.01–2.74, and 3.98–5.05 times than those in AC142 leaves, respectively (**Figure 6B**). These results strongly demonstrated that *StMYBA1* could promote the accumulation of anthocyanin in potato leaves. The regulatory role of *StMYBA1* was further investigated by analyzing the expression of the structural genes. The results showed that overexpression of the *StMYBA1* gene could increase the expression of structural genes (*StCHS*, *StCHI*, *StF3H*, *StF3’H*, *StF3’5’H*, *StDFR*, *StANS*, and *StGST*) significantly (**Figures 6C–K**), implying that the *StMYBA1* gene promotes anthocyanin biosynthesis in potato leaves by upregulating the expression of structural genes.

## 4 Discussion

Anthocyanins not only have a strong antioxidant capacity, but also can protect plants against various biotic and abiotic stresses (Sivankalyani et al., 2016; Liu et al., 2019). The structural genes in the anthocyanin synthesis pathway are mainly regulated by the members of the MBW complex (Lloyd et al., 2017; Cui et al., 2021), especially the MYB TFs (Lai et al., 2013). In this study, the transcriptome analysis of light sensitive tuber samples in potato showed that the expression level of a MYB gene, *StMYBA1*, gradually increased during the anthocyanin biosynthesis (**Figure 4B**, **Table S1**), and the promoter activity of the *StMYBA1* in light conditions was significantly higher than that in dark (**Figure 5B**). These results indicated that *StMYBA1* gene was a light-induced transcription factor associated with anthocyanin biosynthesis. Previous studies also showed that the promotion of anthocyanin biosynthesis in tobacco leaves by *StMYBA1* gene was light dependent (Liu et al., 2017). MYB genes which regulate anthocyanin biosynthesis induced by light also exist in many species such as *Arabidopsis*, apple, and so on (Maier et al., 2013; Yao et al., 2020). When dark-grown fruit was exposed to sunlight, transcript levels of *MdMYB1* in apple increased correlating with anthocyanin biosynthesis in the skin (Takos et al., 2006).

**Figure 7:**
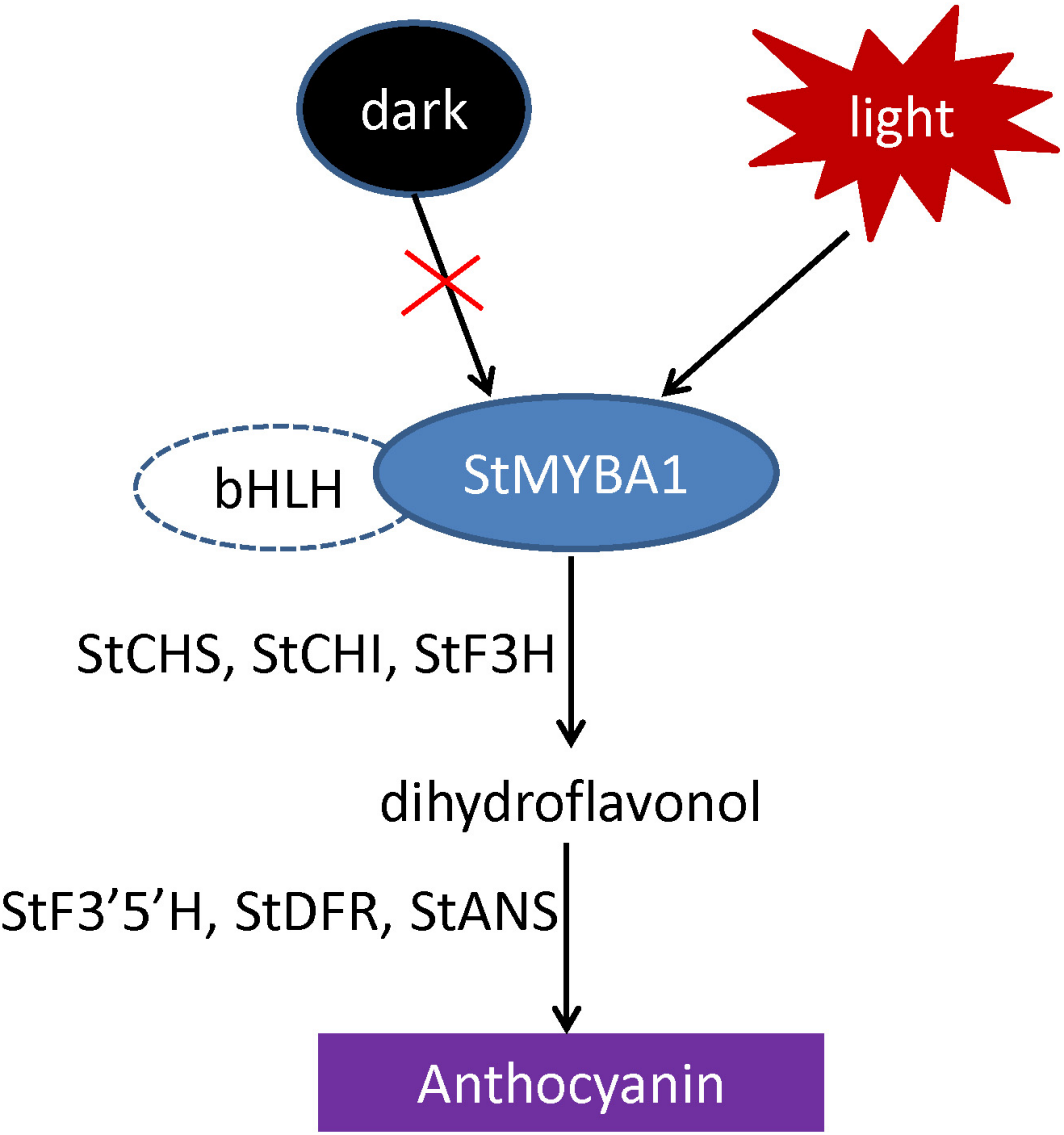
The regulatory model of *StMYBA1* gene on potato anthocyanin biosynthesis.

So far, studies on structural genes of potato anthocyanin biosynthesis mainly focuses on the late synthesis genes, such as *StF3’5’H*, *StDFR*, *StANS* and *3GT* (Jung et al., 2005; Zhang et al., 2009; Zhang et al., 2020). In this study, the expression level of the light-induced *StMYBA1* was highly correlated with the expression trend of early synthesis genes *CHS* and *CHI* (**Figure 4**), and the promoter activity of early synthesis genes *StCHS*, *StCHI*, and *StF3H* could be activated by StMYBA1 under light conditions (**Figure 5**), these results showed that *StMYBA1* has a direct regulatory effect on late synthetic genes. Further gene function analysis found that the anthocyanin accumulation and expression of structural genes in transgenic leaves that overexpressed *StMYBA1* was significantly higher than that of the control (**Figure 6**). All of these results demonstrate that the *StMYBA1* gene promoted anthocyanins biosynthesis in potato by activating the expression of structural genes under light conditions (**Figure 7**). These findings provide a theoretical basis and genetic resources for the regulatory mechanism of anthocyanin biosynthesis in potato.

StMYBA1 can activate the expression of early biosynthetic genes of anthocyanin biosynthesis *StCHS*, *StCHI*, and *StF3H*, while the expression of late synthesis gene *StF3’5’H* has no significant difference in the early stage of light induction, and its expression increases rapidly at 48 hours (**Table S2**). It is speculated that StMYBA1 activates the expression of the early synthesis gene, resulting in the accumulation of dihydrokaempferol, and further generating delphinidin from dihydrokaempferol with the participation of StF3’5’H. The expression of the late synthetic gene *StF3’H* increased at 6 hours of light induction, and then the expression was relatively stable (**Table S2**), but the purple anthocyanin cyanidin controlled by StF3’H did not accumulate. This indicates that the anthocyanins accumulation in tubers induced by light requires the regulation of other transcription factors on late synthetic genes besides the *StMYBA1* gene. Therefore, the transcription factors in cluster 3 which are highly correlated with the expression of *StF3’5’H* may be involved in the regulation of anthocyanin biosynthesis. Previous studies showed that the *StWRKY13* gene (Soltu.DM.10G023760.1) and *StbHLH1* (Soltu.DM.09G019660.1) gene in cluster 3 could indeed regulate anthocyanin biosynthesis in potato (Liu et al., 2016; Zhang et al., 2022).

During light induction, RM-210 tuber peel turned into purple gradually, and anthocyanin content showed that only delphinidin content increased, but other components did not (**Figure 1** and **Figure S1**). This is consistent with the results in apple, which showed low intensity would change the color of apple peel, only the content of cyanidin increased. The content of other anthocyanins did not change (Chen et al., 2019). Most of the key genes in transcriptome data are mined using multiple analysis methods (Qi et al., 2021; Zheng et al., 2021). In this study, we used the similar approach. The co-expression cluster analysis indicated that the expression of *StMYBA1* strongly correlated with that of the structural genes *StCHS* and *StCHI* (**Figure 4B**). Therefore, it is speculated that the *StMYBA1* gene may be the key factor in regulating light-induced anthocyanin synthesis in potato.

Different from RM-210 tubers, the contents of pelargonidin and cyanidin in potato leaves overexpressing *StMYBA1* also increased significantly (**Figure 6B**). It may be that the different genetic backgrounds of RM-210 and AC142 lead to the different regulation modes of *StMYBA1*. The promoter activity of late synthetic genes could not be activated by StMYBA1, but their expression in transgenic leaves increased (**Figures 6H–K**), indicating that StMYBA1 is an indirect regulator of late synthetic genes or might need to be regulated together with bHLH in the form of complexes. Previous studies also showed that most MYB genes regulate anthocyanin biosynthesis in the form of complexes (Liu et al., 2016; Liu et al., 2021; Qin et al., 2022). However, the specific regulation mode of the *StMYBA1* gene needs to be further studied.

## Data availability statement

The data presented in the study are deposited in the Harvard Dataverse repository, and the original fpkm value of RNA-seq can be obtained through the website https://dataverse.harvard.edu/dataset.xhtml?persistentId=doi:10.7910/DVN/PYOK2T.

## Author contributions

BS and CH designed the research. SL performed anthocyanin content determination. YZ performed transient assays. XZ performed the potato transformation. XH and TL analyzed the data. XZ analyzed gene expression. XZ, HZ and YL wrote the article. All authors contributed to the article and approved the submitted version.

## Supplementary material

Supplementary Figure 1 The HPLC chromatograms of 6 anthocyanins in RM-210 tubers.

Supplementary Figure 2 Quantitative analysis of transcript levels of differentially expressed genes (DEGs) in RM-210 tubers light-induced. Each column represents the mean value ± standard error (n = 3).

Supplementary Figure 3 Screening of StMYBA1-overexpressed transgenic lines. **(A)** Regeneration bud from the microtuber of potato. **(B)** Rooting screening of transgenic lines. **(C)** The relative expression of the *StMYBA1* gene in the transgenic lines. Each column represents the mean value ± standard error (n = 3). **(D)** Growth status of transgenic plants and the untransformed control. Bars=2 cm.
