# Supplementary figures and images for: Transcriptome analysis provides StMYBA1 gene that regulates potato anthocyanin biosynthesis by activating structural genes
Source: Front Plant Sci. 2023 Jan 20;14:1087121. doi: 10.3389/fpls.2023.1087121 (PMC9895859; doi:10.3389/fpls.2023.1087121)

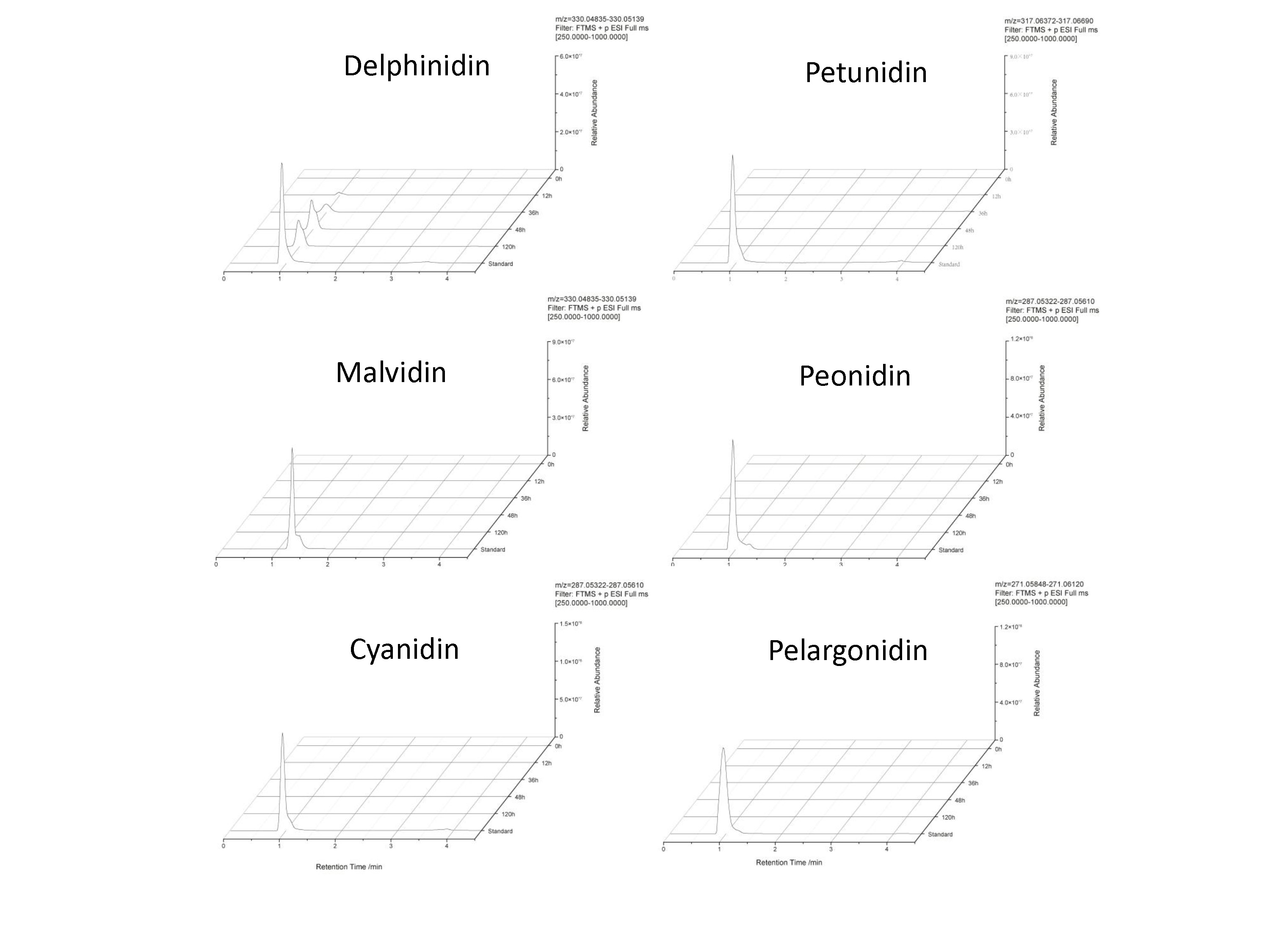

Supplement: Supplementary Figure 1 — The HPLC chromatograms of 6 anthocyanins in RM-210 tubers. [file Image_1.jpeg]

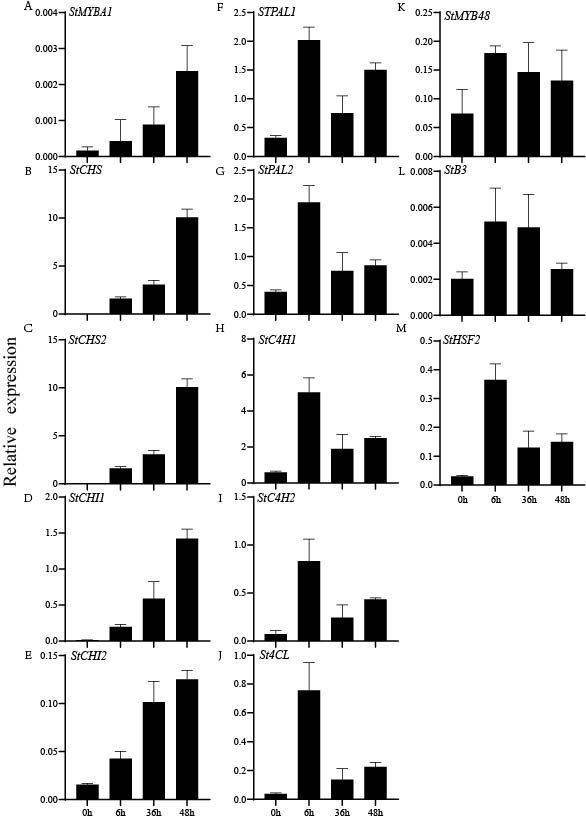

Supplement: Supplementary Figure 2 — Quantitative analysis of transcript levels of differentially expressed genes (DEGs) in RM-210 tubers light-induced. Each column represents the mean value ± standard error (n = 3). [file Image_2.jpeg]

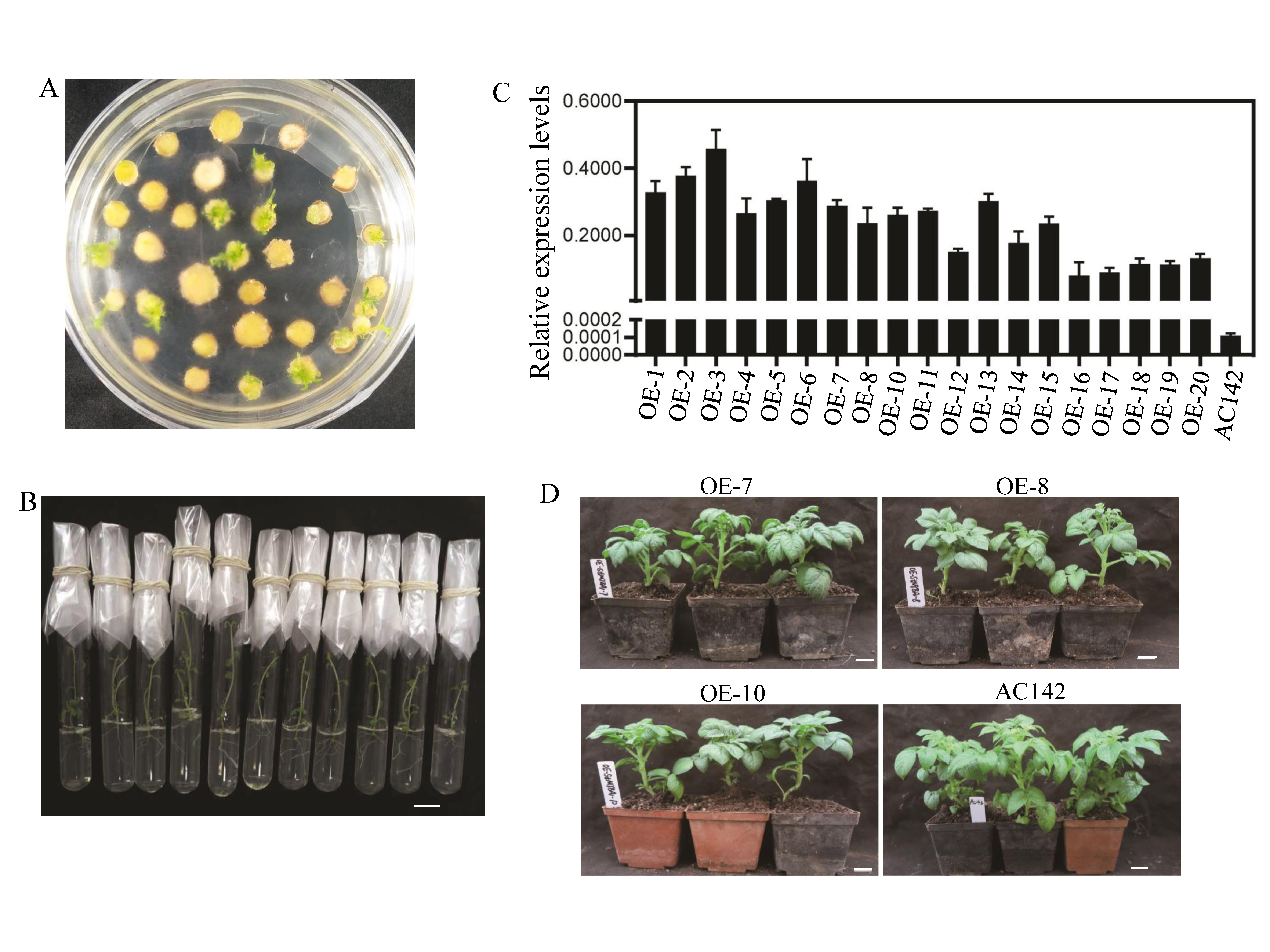

Supplement: Supplementary Figure 3 — Screening of StMYBA1-overexpressed transgenic lines. (A) Regeneration bud from the microtuber of potato. (B) Rooting screening of transgenic lines. (C) The relative expression of the StMYBA1 gene in the transgenic lines. Each column represents the mean value ± standard error (n = 3). (D) Growth status of transgenic plants and the untransformed control. Bars=2 cm. [file Image_3.jpeg]
